# Job vacancy at the European Centre for Disease Prevention and Control

**DOI:** 10.2807/1560-7917.ES.2018.23.41.1810111

**Published:** 2018-10-11

**Authors:** Eurosurveillance editorial team

**Affiliations:** 1 European Centre for Disease Prevention and Control (ECDC), Stockholm, Sweden

The European Centre for Disease Prevention and Control (ECDC) invites applications for the following position:

**Expert Data Management and Data Architecture**

The application deadline expires on 04 Nov 2018. For more information, please visit the ECDC website: https://ecdc.europa.eu/en/work-us/vacancies?f%5B0%5D=deadline_date%3A1

